# Supplementary figures and images for: The role of obesity in physiological stress, balance, and proprioception during repetitive manual material handling tasks
Source: PLoS One. 2025 May 29;20(5):e0324996. doi: 10.1371/journal.pone.0324996 (PMC12121826; doi:10.1371/journal.pone.0324996)

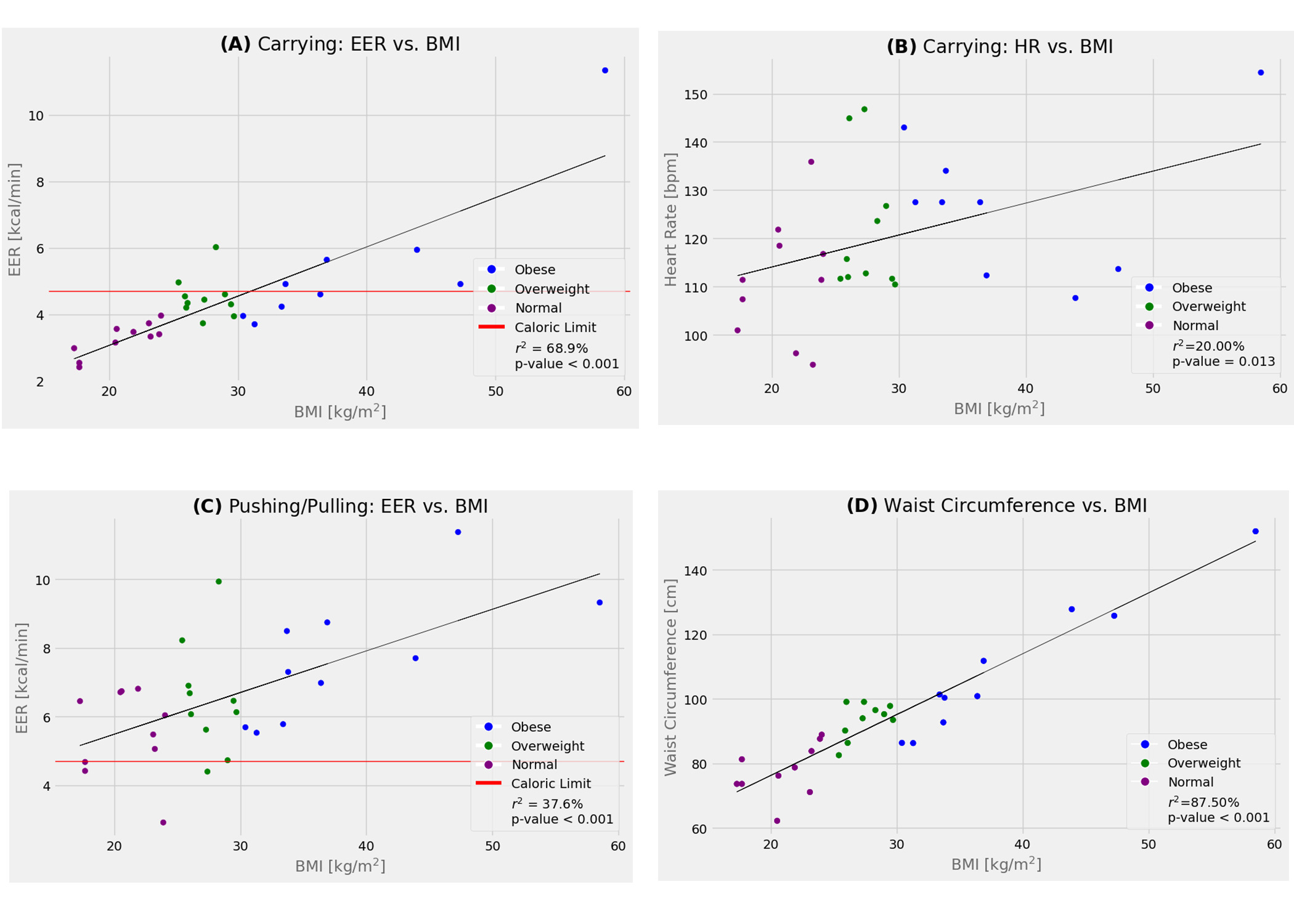

Supplement: S1 Fig — Each circle represents a participant, with colors indicating BMI classification: blue for obese, green for overweight, and purple for normal. The solid red line red represents the 4.7 kcal/min NIOSH threshold. r² and p-values are provided for each graph. The figure represents the relationship between a subject’s BMI and the following variables: (A) Carrying EER; (B) Carrying HR; (C) Pushing/pulling EER; and (D) Waist circumference. (TIF) [file pone.0324996.s001.tif]
