# Supplementary material for: The role of obesity in physiological stress, balance, and proprioception during repetitive manual material handling tasks
Source: PLoS One. 2025 May 29;20(5):e0324996. doi: 10.1371/journal.pone.0324996 (PMC12121826; doi:10.1371/journal.pone.0324996)
Supplement: Attachment — Submitted filename: Response to Reviewers.docx [file pone.0324996.s004.docx]

**Responses to Reviewers’ Comments**

**Reviewer: Dr. Anees Alyafei**

**Review Comments to the Author**

**Reviewer #1: The manuscript presents a technically sound study on the effects of obesity on physiological stress, balance, and proprioception during manual material handling tasks. The methodology is rigorous, and the statistical analysis is appropriate, though the small sample size (n=30) and reliance on BMI as an obesity measure may limit generalizability. Expanding the sample size, incorporating direct body composition assessments, and recruiting occupation-specific participants would enhance validity. The study is clearly written, with well-structured arguments and practical implications for workplace ergonomics. Minor revisions are recommended to improve external applicability and strengthen conclusions.**

*Response:* We sincerely appreciate the reviewer's insightful comments and valuable suggestions. In response to his recommendations, we have revised the manuscript accordingly. Please find our detailed responses below, with revisions highlighted in yellow.

In determining the total number of participants for this study (30 participants), a power analysis was conducted to ensure statistical robustness and meaningful results. This consideration has been incorporated into the Discussion section. Changes have been highlighted in lines 344-349: “A total of 30 participants (15 male and 15 female) were evaluated in the present study. While a larger sample size would have been preferable, results from the post-hoc power analysis indicated that the sample was sufficient to detect significant differences in EER and HR (power ≥ 95%) across BMI groups and sex. Additionally, the sample was adequate for detecting effects in balance/proprioception values (power ≥ 91.3%), considering EER and BMI as the main effects.”

Regarding the incorporation of direct body composition assessment, the limitations of BMI and the use of waist circumference as a substitute were addressed in the Discussion section. These changes have been added and highlighted in lines 352-358: “Furthermore, BMI does not account for body fat percentage or its distribution. Although the researchers selected only obese participants with minimal to no reported exercise history, body fat percentage, which is a more precise indicator of physiological stress than BMI, was not directly measured. However, as stated in the Results section, waist circumference showed an almost perfect correlation with BMI, suggesting that the participants' BMI in this study is closely related to their body fat percentage (Bozeman et al. 2012) and can be used as a substitute.”

Lastly, the limitation of recruiting occupation-specific participant was acknowledged in the Discussion section. Changes have been added and highlighted in lines 349-352: “In addition, the participants' job roles did not consistently involve MMH tasks. Future research should specifically examine the effects of physiological stress, balance, and proprioception on participants that perform MMH tasks as part of their occupation to draw more definitive conclusions.”
