# Supplementary material for: The role of obesity in physiological stress, balance, and proprioception during repetitive manual material handling tasks
Source: PLoS One. 2025 May 29;20(5):e0324996. doi: 10.1371/journal.pone.0324996 (PMC12121826; doi:10.1371/journal.pone.0324996)
Supplement: Attachment — Submitted filename: Response_to_Reviewers_auresp_2.docx [file pone.0324996.s005.docx]

**Responses to Reviewers’ Comments**

**Reviewers: Anees Alyafei, Reviewer #2**

**Review Comments to the Author**

**Reviewer #1:**

**The study employs a rigorous experimental design, including controlled testing conditions for balance, proprioception, and physiological stress. Appropriate statistical methods (two-way ANOVA, mixed-effects models) were used to compare groups. Energy Expenditure Rate (EER) and Heart Rate (HR) as physiological markers are well-chosen and valid indicators of fatigue and stress. The study follows ethical research guidelines, with Institutional Review Board (IRB) approval and informed consent obtained.**

**Small Sample Size (n=30):**

**Although a post-hoc power analysis justifies the sample size, a larger sample would improve generalizability. BMI is used as the primary obesity classification metric. While the authors include waist circumference, more precise.**

*Response:* We sincerely appreciate the reviewer's feedback and valuable suggestions. In response to his recommendations, we have revised the manuscript accordingly. Please find our detailed responses below, with revisions highlighted in yellow.

Regarding the sample size of 30 participants, we acknowledged this as a limitation in the Discussion section, mentioning the inclusion of a post-hoc power analysis and that a larger sample size would be ideal. This was included and highlighted in lines 342-347: “A total of 30 participants (15 male and 15 female) were evaluated in the present study. While a larger sample size would have been preferable, results from the post-hoc power analysis indicated that the sample was sufficient to detect significant differences in EER and HR (power ≥ 95%) across BMI groups and sex. Additionally, the sample was adequate for detecting effects in balance/proprioception values (power ≥ 91.3%), considering EER and BMI as the main effects.”

**Long and Complex Sentence Structures:**

**Original: "Manual laborers are prone to fatigue-related incidents, increasing the risk of balance disturbances and falls. Our previous work showed that obese individuals can reach critical fatigue levels during repetitive lifting, as defined by National Institute for Occupational Safety and Health (NIOSH). Therefore, assessing fatigue levels during manual handling activities beyond lifting is crucial to determine if this body mass index (BMI)-based pattern persists."**

**Issue: The sentence is long and contains multiple ideas that could be broken down for clarity.**

**Suggested Revision: "Manual laborers often experience fatigue-related incidents, which increase their risk of balance disturbances and falls. Previous research indicates that obese individuals may reach critical fatigue levels during repetitive lifting. This study examines whether this BMI-based fatigue pattern also applies to other manual handling activities."**

*Response:* We have addressed it as suggested and incorporated the following revisions in lines 002–005: “Manual laborers often experience fatigue-related incidents, which increase their risk of balance disturbances and falls. Previous research indicates that obese individuals may reach critical fatigue levels during repetitive lifting. This study examines whether this BMI-based fatigue pattern also applies to other manual handling activities.”

**Original: "Additionally, evaluating balance loss when physiological stress becomes critical will help quantify the increased fall risk for obese individuals."**

**Issue: The phrase "evaluating balance loss when physiological stress becomes critical" is somewhat ambiguous.**

**Suggested Revision: "Assessing balance impairment under high physiological stress conditions will help quantify the increased fall risk in obese individuals."**

*Response:* Suggested revisions have been added and highlighted in lines 005-007: “Therefore, assessing balance impairment under high physiological stress conditions will help quantify the increased fall risk in obese individuals.”

**Minor Typographical and Grammatical Errors:**

**Original: "Results indicated a positive correlation between BMI and EER for both carrying (p = 0.003) and pushing/pulling (p = 0.013). In the mixed-effects model, BMI (p = 0.032) and EER (p = 0.037) were positively correlated with knee proprioception loss, whereas EER was negatively correlated with balance (p = 0.020). These findings confirm that obese individuals face a critical fatigues levels and impaired proprioception and balance during repetitive handling tasks."**

**Issue: "face a critical fatigues levels" contains a grammatical error ("fatigues" should be "fatigue").

Suggested Revision: "These findings confirm that obese individuals face critical fatigue levels, as well as impaired proprioception and balance, during repetitive handling tasks."**

*Response:* We have corrected this sentence and highlighted the changes in lines 014–016: “These findings confirm that obese individuals face critical fatigue levels, as well as impaired proprioception and balance, during repetitive handling tasks.”

**Original: "Carrying and pushing/pulling tasks were performed by 30 participants, with weights calculated using the Liberty Mutual Equations to meet NIOSH criteria."

Issue: "Carrying and pushing/pulling tasks were performed by 30 participants" is slightly awkward in construction.**

**Suggested Revision: "Thirty participants performed carrying and pushing/pulling tasks, with weights determined using the Liberty Mutual Equations to align with NIOSH criteria.**

*Response:* Suggested revision was added and highlighted in lines 007–008: “Thirty participants performed carrying and pushing/pulling tasks, with weights determined using the Liberty Mutual Equations to align with NIOSH criteria.”

**Reviewer #2: (No Response)**
